# Uncertainty Prediction for Monocular 3D Object Detection

**DOI:** 10.3390/s23125395

**Published:** 2023-06-07

**Authors:** Junghwan Mun, Hyukdoo Choi

**Affiliations:** Department of Electronic Materials, Devices, and Equipment Engineering, Soonchunhyang University, Asan 31538, Republic of Korea; wjdghks511@sch.ac.kr

**Keywords:** uncertainty estimation, uncertainty evaluation, object detection, deep learning, self-driving

## Abstract

For object detection, capturing the scale of uncertainty is as important as accurate localization. Without understanding uncertainties, self-driving vehicles cannot plan a safe path. Many studies have focused on improving object detection, but relatively little attention has been paid to uncertainty estimation. We present an uncertainty model to predict the standard deviation of bounding box parameters for a monocular 3D object detection model. The uncertainty model is a small, multi-layer perceptron (MLP) that is trained to predict uncertainty for each detected object. In addition, we observe that occlusion information helps predict uncertainty accurately. A new monocular detection model is designed to classify occlusion levels as well as to detect objects. An input vector to the uncertainty model contains bounding box parameters, class probabilities, and occlusion probabilities. To validate predicted uncertainties, actual uncertainties are estimated at the specific predicted uncertainties. The accuracy of the predicted values is evaluated using these estimated actual values. We find that the mean uncertainty error is reduced by 7.1% using the occlusion information. The uncertainty model directly estimates total uncertainty at the absolute scale, which is critical to self-driving systems. Our approach is validated through the KITTI object detection benchmark.

## 1. Introduction

In this paper, we present a novel method to address the problem of uncertainty in 3D object detection using monocular cameras in a road environment. Object detection plays a critical role in the perception system of self-driving vehicles, providing essential information for path planning and ensuring safe and efficient vehicle operation. Significant progress has been made in 2D object detection [1,2,3,4,5,6], but the challenges of detecting objects in 3D space [7,8,9,10,11,12,13,14,15] are significantly greater, especially because of the complexity of accurately representing 3D bounding boxes.

Our research focuses on predicting the uncertainty associated with 3D object detection results. Most previous studies have focused on improving detection metrics using benchmarks such as KITTI [16] and NuScenes [17], while the discussion of the uncertainty in predicted bounding boxes has remained limited. It is important to understand that even if the intersection over union (IoU) between the predicted and ground truth (GT) bounding boxes exceeds a certain threshold, there is always some degree of error or uncertainty in the predicted box parameters. For autonomous vehicles to plan safe routes, it is important to understand the uncertainty of the bounding boxes, which can help set appropriate safety margins. Unlike previous works, our method does not require repetitive inferences or complex mathematical derivations [18,19,20,21,22], but just a small additional model is adopted to predict uncertainty.

To address this challenge, we propose a novel uncertainty prediction model based on neural networks. The key feature of our model is its ability to predict uncertainty on an absolute scale. We describe in detail how we generate the training data and make the necessary adjustments to improve the accuracy of the predictions. In addition, we use occlusion information to improve the accuracy of uncertainty estimation. Since occluded objects are difficult to infer accurately, incorporating occlusion levels into uncertainty prediction becomes crucial.

We propose a method to evaluate the accuracy of uncertainty prediction by statistically analyzing the error values of the bounding box predictions. Uncertainty accuracy is a concept that measures how closely the predicted uncertainty value matches the actual level of uncertainty. Uncertainties are evaluated using standard deviations, assuming that the error values follow a normal distribution.

To evaluate our method, we utilized the KITTI dataset [16], a widely used benchmark in the field of autonomous driving. Our tests found an average discrepancy of 4.92% in the predicted standard deviation values.

The key contributions of this paper can be summarized as follows. First, we propose a neural network–based uncertainty prediction model that operates on an absolute scale. We demonstrate how to prepare training data and adjust predictions. Second, occlusion information is used to improve the accuracy of uncertainty estimation. Finally, we introduce a statistical analysis method to evaluate the accuracy of uncertainty prediction. By addressing the uncertainty in 3D object detection, our proposed methodology enables self-driving vehicles to operate more safely and efficiently.

## 2. Preliminaries

Our work is mainly based on two themes: 3D object detection and uncertainty estimation. The following subsections introduce the recent studies in these fields.

### 2.1. 3D Object Detection

Deep learning approaches to 3D object detection have been studied extensively and typically rely on lidar, cameras, or some combination of the two. The performances of the methods based on lidar or stereo cameras have been greatly improved, but monocular approaches still face challenges. We categorize the previous works by sensor type and review them one by one.

(1) 3D Object Detection via Lidar: Lidar provides 3D geometric information in the form of point clouds. Most detector models are based on a CNN, but irregular point clouds are not fit for CNNs. To regularize the data format, point clouds are encoded in regular 2D-grid or 3D-voxel space. Various methods based on 3D convolution [7,8,23], bird’s eye view (BEV) images [9,24], and point pillars [25] have been proposed. CenterPoint [26] employs a two-stage detector architecture on BEV images. The backbone and region proposal network (RPN) output dense predictions of class confidence and bounding box parameters without anchors, and then the MLP head takes the features from the five points of the predicted bounding box as input to refine the results. PointPainting [27] fuses image information into the existing lidar-based detectors. The semantic segmentation result from an image can be used to enrich the information of a point cloud. A semantically labeled point cloud can be input to existing models, such as PointPillars [25], VoxelNet [7], and PointRCNN [23], with improved results.
(2) 3D Object Detection via Stereo Cameras: Depth information can be extracted from stereo images, given that disparities are accurately estimated. However, as the quality of the depth data is not as good as with lidar, point clouds are not generally extracted from stereo images for 3D object detectors. Instead, stereo information is merged at the feature level [10,11,28].

A stereo R-CNN [10] concatenates the features from stereo images to produce left and right regions of interest (RoIs) simultaneously. Four key points are predicted for each object from a 2D image, and a 3D box is estimated from the key points. The final depth of an object is adjusted by minimizing the photometric reprojection error between the left and right RoIs. A deep stereo geometry network (DSGN) [11] utilizes 3D volumetric representations. Left and right features are merged to make a plane-sweep volume (PSV) in camera frustum space, which is warped to a 3D geometry volume (3DGV). The 3DGV is squeezed out to a 2D feature map, and it is input to the BEV-based detector.

(3) 3D Object Detection via Monocular Cameras: Since a monocular camera provides no depth information, monocular 3D detection is a challenging task. To overcome this difficulty, some monocular approaches have exploited depth map prediction [12,13,14,29]. M3D-RPN [13] is an anchor-based detector that uses 2D and 3D anchors. The key feature is the depth-ware convolution, which trains different kernels for row-wise separated blocks to exploit the fixed-view assumption of self-driving vehicles. SMOKE [29] is a single-stage 3D detector that classifies the keypoint heatmap of projected 3D object centers and predicts bounding box parameters at the keypoints. MonoFlex [12] model specializes in detecting edge-truncated objects. It decouples the learning process of inside objects and truncated objects. Depths are directly predicted by the model for each object and estimated from the keypoint predictions simultaneously. The two depth predictions are merged based on the uncertainties.

### 2.2. Uncertainty Estimation

Bayesian neural networks (BNNs) [18] are devised to model the uncertainty of neural networks. It is assumed that each weight has a probabilistic distribution instead of a deterministic value. It begins with the a priori distribution and updates the posterior distribution through training. It is known to restrain an overfitting problem, but inferring the output posterior in a deep network is computationally expensive. To tackle this problem, Bayesian approximation using dropout techniques [19] is proposed. Model output uncertainty is captured using Monte Carlo (MC) sampling, inferring from the same input multiple times with dropout. It can be applied to existing networks and is relatively efficient. Based on the MC sampling approach, F. Di et al. [20] proposed a method to capture uncertainty for a lidar-based 3D object detector. The output uncertainty is divided into epistemic uncertainty and aleatoric uncertainty, presenting model uncertainty and observation noises from sensors. It quantifies classification uncertainty through Shannon entropy and mutual information and regression uncertainty based on total variance. F. Kraus and K. Dietmayer [21] applied this approach to the 2D one-stage detector YOLOv3 [5]. A. Loquercio et al. [22] presented a method to analyze uncertainty from already-trained models without changing the optimization process. Model uncertainty is estimated from MC sampling, whereas data uncertainty is estimated using assumed density filtering (ADF), which analytically propagates initial sensor noise to the output.

Another approach is to explicitly output distribution parameters from networks. Gaussian YOLOv3 [6] predicts the mean and variance of bounding box parameters. It is optimized by minimizing the log-likelihood of GT parameters from the predicted distribution.

Although the existing methods have demonstrated their effectiveness in various ways, they have some weaknesses. MC sampling requires multiple inferences, thus slowing the effective model speed, and predicts uncertainty only in relative scales, not in the absolute scale. To calculate safe margins for driving vehicles, uncertainty information should be scale-aware. Predicting distribution parameters can possibly learn absolute scale, but it needs to change the head structure and loss function and thus is not applicable to the existing models. In contrast, we train an independent network to estimate bounding box uncertainty after the detector model is completely trained. Our approach is applicable to already-trained models at little additional cost.

## 3. Uncertainty Prediction Model

**Figure 1:**
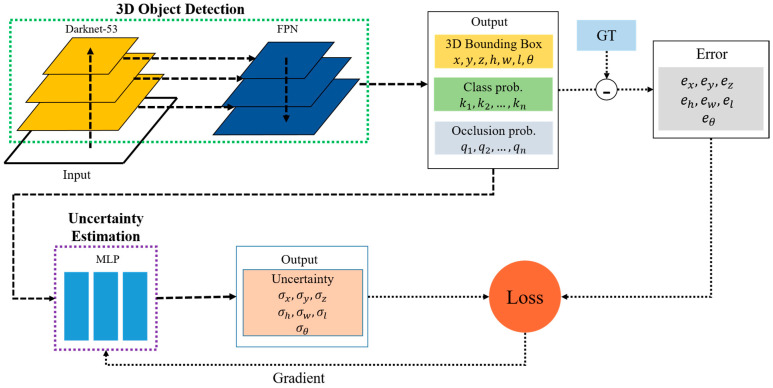
Overview of the system.

Our goal is to predict uncertainties of bounding box parameters using a trainable model. The proposed system is depicted schematically in Figure 1. The 3D object detection model predicts occlusion probabilities as well as bounding boxes and class probabilities. Bounding box errors are obtained by comparing the detection results with the annotated ground truth (GT).

The detection results are input to the uncertainty model, and it predicts the standard deviations of the bounding box parameters. The outputs are trained by the bounding box errors. The key feature of our system is to utilize occlusion-level information, integrating this critical aspect into the uncertainty prediction. The details of the models are described in the following sections. Table 1 describes the nomenclature used in the rest of the paper.

### 3.1. Monocular 3D Object Detector

Since our aim is to train the uncertainty model using occlusion information, a custom detection model has to be trained rather than using the existing models. We verify that occlusion is relevant to the bounding box uncertainty in the experiment section. Our detection model architecture is based on YOLOv3 [5]. A monocular image is input to the model, and the output composition is modified to predict 3D bounding boxes and occlusion levels. The *i*th output instance $X_{i}$ contains the following parameters:

(1) $$X_{i}=\left[\begin{array}{cccccccccccccccccc} y_{i}^{\prime } & x_{i}^{\prime } & h_{i}^{\prime } & w_{i}^{\prime } & y_{i} & x_{i} & z_{i} & l_{i} & w_{i} & h_{i} & \theta_{i} & o_{i} & c_{i1} & \ldots & c_{iK} & u_{i1} & \ldots & u_{iM} \end{array}\right]$$

which is comprised of coordinates $\left(y_{i}^{\prime },x_{i}^{\prime }\right)$ and dimensions $\left(h_{i}^{\prime },w_{i}^{\prime }\right)$ of a 2D bounding box, coordinates $\left(y_{i},x_{i},z_{i}\right)$, dimensions $\left(l_{i},w_{i},h_{i}\right)$ and a yaw angle $\left(\theta_{i}\right)$ of a 3D bounding box, an objectness $\left(o_{i}\right)$, class probabilities $\left(c_{i1},\ldots ,c_{iK}\right)$, and occlusion-level probabilities $\left(u_{i1},\ldots ,u_{iM}\right)$ where there are K object classes and M occlusion levels.

During training, the parameters up to $\theta_{i}$ are trained using the L1 smooth loss, and the rest of the parameters are trained using the cross-entropy loss. During inferencing, the non-maximal suppression (NMS) algorithm is applied to the 2D bounding boxes in advance, and then the 3D box NMS is applied to the detected 2D objects. The activation functions for bounding box parameters are similar to those of M3D-RPN. However, as our detector does not use anchors, the raw depth prediction $t_{z,i}$ is activated by Equation (2).

(2) $$z_{i}=10\exp\left(t_{z,i}\right)$$

### 3.2. Uncertainty Model

The uncertainty model predicts the standard deviation of error in 3D bounding box parameters from $x_{i}$ to $\theta_{i}$. To achieve this, the model takes the related parameters from the detected instances, which are 3D bounding box parameters and occlusion-level probabilities, as defined in Equation (3).

(3) $$Y_{i}=\left[\begin{array}{ccccccccccccc} y_{i} & x_{i} & z_{i} & l_{i} & w_{i} & h_{i} & \theta_{i} & c_{i1} & \ldots & c_{iM} & u_{i1} & \ldots & u_{iM} \end{array}\right]$$

The model architecture is an MLP model with three hidden layers, each comprising 64 channels, suitable for the low-dimensional input data. The model outputs the standard deviations for bounding box parameters per the following equation.

(4) $$Z_{i}=\left[\begin{array}{ccccccc} \sigma_{y,i} & \sigma_{x,i} & \sigma_{z,i} & \sigma_{l,i} & \sigma_{w,i} & \sigma_{h,i} & \sigma_{\theta ,i} \end{array}\right]$$

To create label data for the uncertainty model, the optimal standard deviation should be deduced for a detected instance. For training data, bounding box error can be calculated from the GT bounding box data. Although statistical data cannot be derived from a single datum, we need the standard deviation from a single error value. If the probabilistic density function is parameterized by the standard deviation with a given error, as defined in Equation (5), the optimal standard deviation is derived by finding the value that maximizes the probability in Equation (6).

(5) $$p\left(\sigma_{x}\right)=\frac{1}{\sqrt{2\pi }\sigma_{x}}\exp\left(-\frac{e_{x^{2}}}{\sigma_{x^{2}}}\right)$$

(6) $$\frac{\partial p\left(\sigma_{x}\right)}{\partial \sigma_{x}}\;=-\frac{1}{\sqrt{2\pi }\sigma_{x^{2}}}\exp\left(-\frac{e_{x^{2}}}{\sigma_{x^{2}}}\right)+\frac{1}{\sqrt{2\pi }\sigma_{x}}\exp\left(-\frac{e_{x^{2}}}{\sigma_{x^{2}}}\right)\frac{2e_{x^{2}}}{\sigma_{x^{3}}}\;=\frac{1}{\sqrt{2\pi }\sigma_{x^{2}}}\exp\left(-\frac{e_{x^{2}}}{\sigma_{x^{2}}}\right)\left(-1+\frac{e_{x^{2}}}{\sigma_{x^{2}}}\right)=0$$

The solution is simply $\sigma_{x}=e_{x}$, where x can be replaced by any parameters in a 3D bounding box. Therefore, the uncertainty model is trained to predict the absolute error of 3D bounding box parameters. It is not possible to predict error values, but as a result, the model learns the mean of error in the given situation.

## 4. Uncertainty Evaluation

**Figure 2:**
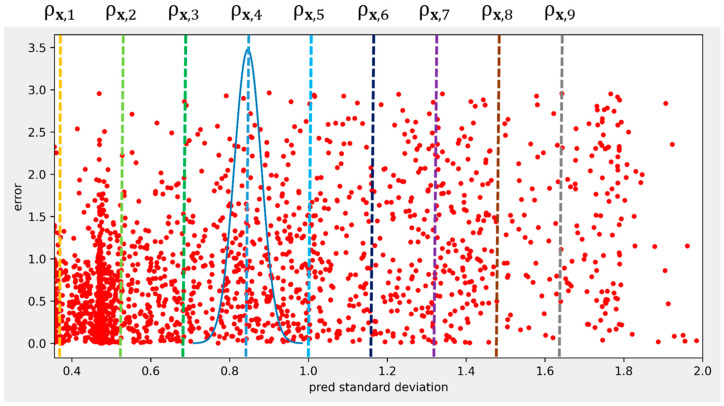
Scatter plot of x coordinate errors against predicted standard deviation. The dotted vertical lines are the sampled standard deviations, and the curve represents Gaussian weights for the fourth sample.

The goal of the uncertainty model is to predict statistical uncertainty, not error. Comparing the model outputs with the corresponding error is not the right way to evaluate the model. Figure 2 is a scatter plot of errors against the predicted standard deviation. To evaluate the accuracy of the predicted standard deviations, the actual standard deviation should be computed using the errors at the specific predicted value. However, as there are few errors at the specific standard deviation, we use errors around the specific standard deviation with Gaussian weights, depicted by the curve in Figure 2. The actual standard deviations are calculated only at the representative sample points rather than at all the predicted standard deviations for computational efficiency.

The sample points are the nine equally spaced standard deviation values between the 10% and 90% quantiles of the predicted standard deviations from the training data, as presented in Equation (7).

(7) $$\rho_{x,n}=\sigma_{x}^{10\%}+\frac{\left(\sigma_{x}^{90\%}-\sigma_{x}^{10\%}\right)}{8}\left(n-1\right),\;\;n=1,\ldots ,9$$

where $\rho_{x,n}$ is the *n*th sample point and $\sigma_{x}^{10\%}$ and $\sigma_{x}^{90\%}$ are the 10% and 90% quantile values, respectively. The sample points are marked by dotted lines in Figure 2. The Gaussian weights are computed from Equation (8).

(8) $$\omega_{x,i,n}=\frac{1}{\sqrt{2\pi }\sigma_{\omega }}\exp\left(-\frac{{\left(\sigma_{x,i}-\rho_{x,n}\right)}^{2}}{\sigma_{\omega }}\right),\;\sigma_{\omega }=\left(\rho_{x,2}-\rho_{x,1}\right)/4$$

where $\omega_{x,i,n}$ is the weight of $\sigma_{x,i}$ to compute the actual standard deviation at the *n*th sample point. At the sample points, the actual standard deviation is computed using all the errors with the Gaussian weights by Equation (9).

(9) $${\hat{\rho }}_{x,n}^{2}={\left(\sum_{i=1}^{L}\omega_{x,i,n}\right)}^{-1}\sum_{n=1}^{N}\omega_{x,i,n}e_{x,i}^{2}$$

where ${\hat{\rho }}_{x,n}$ is the actual standard deviation to be compared with the sampled standard deviation, $\rho_{x,n}$. Ideally, the estimated actual standard deviation is close to the predicted standard deviation at all the sample points, but they are different in reality. Instead, the actual and predicted values are linearly related. To reduce the gap, the predicted standard deviations at the sampling points are adjusted using linear regression, as in Equation (10).

(10) $$\rho_{x,n}^{\prime }=\alpha_{x}\rho_{x,n}+\beta_{x}$$

where coefficients $\alpha_{x}$ and $\beta_{x}$ are optimized by the training data.

Finally, we can assess the accuracy of the predicted uncertainty by comparing the actual value, ${\hat{\rho }}_{x,n}$, with the adjusted prediction, $\rho_{x,n}^{\prime }$. The accuracy of uncertainties is evaluated for all bounding box parameters.

## 5. Experiments

### 5.1. Dataset and Training

In order to train the detection and uncertainty models, we need a variety of labels, including 2D and 3D bounding boxes, object classes, and occlusion levels. The KITTI object detection dataset [16] is the only one meeting these requirements. It provides 7481 frames of labeled data. The dataset is split into training and testing data, similar to the method used in [30]. The input resolution is fixed to 1024 × 320.

The detection model is trained through 140 epochs with a single RTX 3090. Once the detection model training is complete, model inference proceeds with both the training and testing data. The detected objects are matched with the labeled objects using 2D bounding boxes to compute errors for the 3D bounding box parameters. Matching is based on 2D bounding boxes in order to extend the range of 3D bounding box errors without confusing objects. The errors are used as labels to train the uncertainty model. The structure of the uncertainty model is implemented according to Section 3.2, and the Huber loss and Adam optimizer are adopted for training.

Before discussing uncertainty accuracy, we analyze the 3D bounding box errors to verify whether occlusion information is relevant to bounding box uncertainty. The KITTI dataset categorizes objects into three occlusion levels, 0 to 2. A higher occlusion level means that the object is highly occluded. The analysis results are summarized in Table 2. The errors in Level 1 are clearly larger than those in Level 0, and they increase slightly in Level 2. It is proven that occlusion is an important factor for bounding box estimation errors.

### 5.2. Evaluation Results

**Figure 3:**
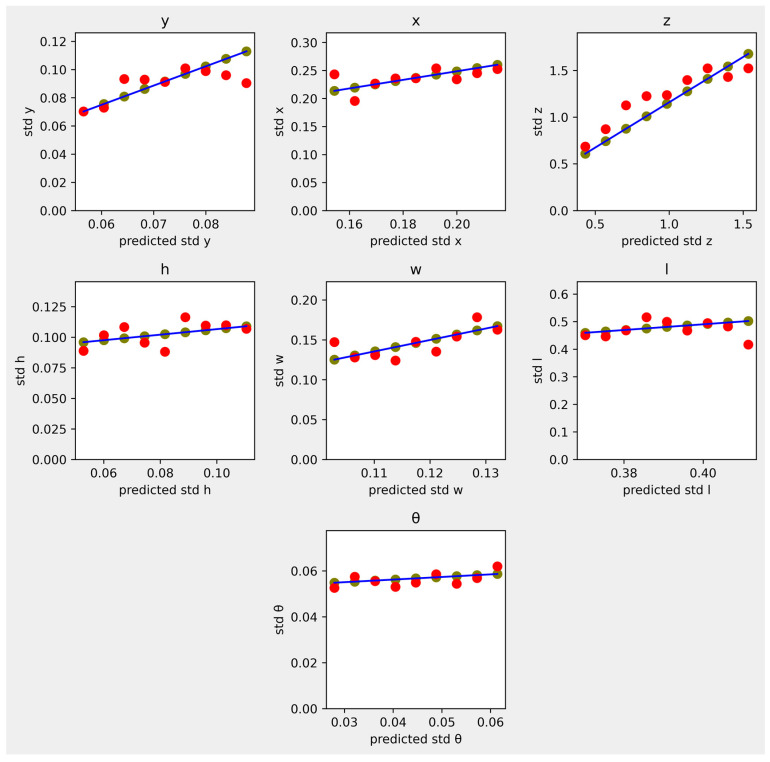
Scatter plot of actual standard deviations against predicted standard deviations for the seven 3D bounding box parameters. The green dots are the adjusted predictions, and the red dots are the actual standard deviation calculated by Equation (9).

We evaluate both the detection and uncertainty models, but our focus is the uncertainty model. The detection model achieves 28.33 AP for the car class in the KITTI 3D object detection benchmark. The standard deviation errors of the uncertainty model are visualized in Figure 3. There are seven subplots of bounding box parameters and nine sample points in each subplot. The equally sampled points are marked by the green dots on the blue line, where the horizontal coordinate is the raw standard deviation prediction and the vertical coordinate is the prediction adjusted by Equation (10). For the red dots in the figure, the vertical axis is the actual standard deviation, calculated using Equation (9). Ideally, the red dots should be located on the blue lines, similar to the green dots, indicating that the prediction is equal to the actual value. We can see that the estimated actual values differ slightly from the predicted values, but they are generally highly correlated.

The numerical results are presented in Table 3. The mean error of the nine sample points and the mean error rates at the sample points are calculated. The four cases are evaluated with different input compositions for the uncertainty model. The worst case comes from the least information. Inference from only bounding box parameters results in the largest error. As more input information is appended, the accuracy improves. In particular, probabilities for occlusion-level classification, denoted as ‘occlusion prob.’ in the table, help reduce error considerably, as we expected. It reduces 7.1% of the mean standard deviation error compared to the result without it. However, occlusion probabilities are available only when the training dataset has occlusion-level labels, as in the KITTI object detection dataset. To generalize this approach, we calculate the occlusion ratio of a 2D bounding box and replace occlusion probabilities with this value. Occlusions are found by checking overlapping 2D bounding boxes, and an object with a greater depth is regarded as occluded. When the occlusion ratio is appended to the input, the result is somewhat improved from the input without it, but it is not as effective as occlusion probabilities trained by manual labels. This means that human labelers evaluate the severity of the occlusion qualitatively more accurately than quantitatively. In addition, occlusion probabilities are learned more effectively using cross entropy loss, while the occlusion ratio is learned using the L1 smooth loss.

It is noteworthy that the z element shows the highest standard deviation errors among all parameters. This is because the absolute value of the z element is generally high, and the monocular camera is not suitable for estimating depths. This is analyzed as the main reason for the low detection accuracy of the monocular approaches.

Some parameters are manually selected in the uncertainty model and evaluation process, and the most impactful parameter is $\sigma_{\omega }$, the standard deviation to compute Gaussian weights in Equation (8). It influences the estimation of the actual standard deviation, ${\hat{\rho }}_{x,n}$. Ideally, $\sigma_{\omega }$ should be as small as possible so that only data close to the selected sample points, $\rho_{x,n}$, are used. However, small $\sigma_{\omega }$ results in losing numerical stability by estimating standard deviation from a small effective number of samples. On the other hand, large $\sigma_{\omega }$ yields numerical stability, but it considers data farther from the sample points to calculate ${\hat{\rho }}_{x,n}$. Table 4 shows the standard deviation errors with different $\sigma_{\omega }$. The default value of $\sigma_{\omega }$ given in Equation (8) is denoted by $\sigma_{\omega }^{\prime }$. Reducing $\sigma_{\omega }^{\prime }$ to one-fourth of its value results in doubling errors on average, and increasing $\sigma_{\omega }^{\prime }$ by a factor of four slightly reduces errors. Larger $\sigma_{\omega }$ results in smaller errors because large $\sigma_{\omega }$ numerically stabilizes the estimation of actual standard deviation, ${\hat{\rho }}_{x,n}$. As a result, the actual values at the sample points are linearly aligned, and thus, it is easy to fit predicted standard deviations to the actual values.

## 6. Conclusions

We have proposed a direct approach to estimating uncertainty at the absolute scale for monocular 3D object detectors. Uncertainties of bounding box parameters are measured as standard deviations and predicted by a simple MLP from the detected object information. We demonstrate how to prepare input and output data to train the uncertainty model. Moreover, in order to evaluate the accuracy of predicted standard deviations, the actual standard deviation is estimated against the specific predicted standard deviation. Although the actual uncertainty is estimated approximately, we can adjust the scale and offset to the predicted uncertainties with training data and evaluate the accuracy of the predicted values with test data using the estimated actual uncertainty. Through the evaluation process, we prove that occlusion information helps improve uncertainty accuracy.

Unlike previous studies, our model does not require repetitive inferences but estimates uncertainty as a standard deviation in a metric unit. Model uncertainty and data uncertainty are not treated separately, but what we need for self-driving is the total uncertainty on a physical scale. Theoretically, standard deviations predicted from our model can be used directly to set a safe margin for the detected objects. Our approach can be extended further to any regression problem, such as various forms of object detection or pose estimation.

## Figures and Tables

**Table 1:** Nomenclature of the uncertainty estimation system.

Symbols	Description
$X_{i}$	*i*th output vector of the detector
$Y_{i}$	*i*th input vector of the uncertainty model
$Z_{i}$	*i*th output vector of the uncertainty model
$\sigma_{x,i}$	predicted standard deviation of parameter **x** in $Z_{i}$
$e_{x,i}$	box prediction error of parameter **x** in $X_{i}$
$\sigma_{x}^{q\%}$	q% quantile over predicted standard deviations
$\rho_{x,n}$	*n*th sample point of parameter **x** from predicted standard deviations
${\hat{\rho }}_{x,n}$	actual standard deviation corresponding to $\rho_{x,n}$
$\omega_{x,i,n}$	weight of $\sigma_{x,i}$ to compute ${\hat{\rho }}_{x,n}$
$\sigma_{\omega }$	standard deviation for the normal distribution of weights
$\rho_{x,n}^{\prime }$	predicted standard deviation adjusted from $\rho_{x,n}$

**Table 2:** The mean of bounding box element errors by occlusion difficulty levels in KITTI dataset.

Parameter	Level 0	Level 1	Level 2
x	0.1706	0.1948	0.2056
y	0.0642	0.0680	0.0706
z	0.6829	0.8690	0.9345
h	0.0682	0.0875	0.0954
w	0.1089	0.1215	0.1260
l	0.3874	0.4141	0.4382
θ	0.0360	0.0473	0.0510

**Table 3:** The mean standard deviation errors and error rates (%) per bounding box element according to input compositions.

Parameter	Box Only	Box + Class Prob.	Box + Class Prob. + Occlusion Prob.	Box + Class Prob. + Occlusion Ratio
x	0.0189/9.10	0.0185/7.75	0.0089/3.65	0.0141/5.55
y	0.0059/8.30	0.0034/4.88	0.0040/3.93	0.0041/4.71
z	0.1269/11.60	0.1196/13.03	0.1203/10.19	0.1277/10.14
h	0.0042/3.83	0.0036/3.63	0.0052/5.46	0.0062/5.76
w	0.0062/4.85	0.0129/8.71	0.0048/3.68	0.0094/6.24
l	0.0260/6.15	0.0159/3.34	0.0183/3.79	0.0184/4.00
θ	0.0015/2.74	0.0022/3.92	0.0022/3.76	0.0036/6.17
Mean	0.0271/6.65	0.0252/6.46	0.0234/4.92	0.0262/6.08

**Table 4:** Sigma error probability/ratio by changing $\sigma_{\omega }$.

Parameter	$\sigma_{w}^{\prime }/4$	$\sigma_{w}^{\prime }$	$4\sigma_{w}^{\prime }$
x	0.0274/12.67	0.0089/3.65	0.0119/5.32
y	0.0097/9.10	0.0040/3.93	0.0042/4.74
z	0.0938/12.70	0.1203/10.19	0.0954/8.79
h	0.0072/6.83	0.0052/5.46	0.0016/1.56
w	0.0156/8.88	0.0048/3.68	0.0066/4.49
l	0.0424/7.53	0.0183/3.79	0.0087/1.75
θ	0.0030/5.36	0.0022/3.76	0.0032/5.46
Mean	0.0286/8.40	0.0234/4.92	0.0199/4.47

## Data Availability

The data presented in this study are available on request from the corresponding author. The data are not publicly available due to high complexity of multi-step processing.
